# A large-scale optical microscopy image dataset of potato tuber for deep learning based plant cell assessment

**DOI:** 10.1038/s41597-020-00706-9

**Published:** 2020-10-27

**Authors:** Sumona Biswas, Shovan Barma

**Affiliations:** Department of Electronics and Communication Engineering, Indian Institute of Information Technology Guwahati, Guwahati, Assam, India

**Keywords:** Agriculture, Plant cell biology

## Abstract

We present a new large-scale three-fold annotated microscopy image dataset, aiming to advance the plant cell biology research by exploring different cell microstructures including cell size and shape, cell wall thickness, intercellular space, etc. in deep learning (DL) framework. This dataset includes 9,811 unstained and 6,127 stained (safranin-o, toluidine blue-o, and lugol’s-iodine) images with three-fold annotation including physical, morphological, and tissue grading based on weight, different section area, and tissue zone respectively. In addition, we prepared ground truth segmentation labels for three different tuber weights. We have validated the pertinence of annotations by performing multi-label cell classification, employing convolutional neural network (CNN), VGG16, for unstained and stained images. The accuracy has been achieved up to 0.94, while, F2-score reaches to 0.92. Furthermore, the ground truth labels have been verified by semantic segmentation algorithm using UNet architecture which presents the mean intersection of union up to 0.70. Hence, the overall results show that the data are very much efficient and could enrich the domain of microscopy plant cell analysis for DL-framework.

## Abstract (summary)

**Table:** 

Measurement(s)	phenotypic annotation • plant cell classification • plant cell segmentation • plant morphology trait • plant cell
Technology Type(s)	machine learning • image segmentation • bright-field microscopy
Factor Type(s)	physical grading of plant tissue • morphological grading of plant tissue • tissue grading of potato tuber images
Sample Characteristic - Organism	Solanum tuberosum

Machine-accessible metadata file describing the reported data: 10.6084/m9.figshare.12957506

## Background & Summary

Microscopy image analysis by employing machine learning (ML) techniques advances the critical understanding of several characteristics of biological cells, ranging from the visualization of biological structures to quantification of phenotypes. In recent years, deep learning (DL) has revolutionized the area of ML, especially, in computer vision technology by evidencing vast technological breakthroughs in several domains of image recognition tasks including object detection, medical and bio-image analysis, and so on. In general, the ML implicates complex statistical techniques on a set of images and its recognition efficiency heavily relies on the handcrafted data features; whereas, the DL processes the raw image data directly and crams the data representation automatically. Indeed, its performance highly depends on the large number of diverse images with accurate and applicable labelling. Following the trend, the DL is emerging as a powerful tool for microscopy image analysis, such as cell segmentation, classification, and detection by exploring the dynamic variety of cells. Moreover, the DL pipeline allows discovering the hidden cell structures, such as single-cell size, number of cells in a given area, cell wall thickness, intercellular space distribution, subcellular components, and its density, etc. from microscopy images by extracting the complex data representation in hierarchical way. Meanwhile, it expressively diminishes the burden of feature engineering in traditional ML.

Certainly, several works have been attempted in cell biology and digital pathology domain to provide quantitative support in automatic diagnosis and prognosis by detecting mitosis, nucleus, cells, and the number of cells from breast cancer^1,2^, brain tumour^3^, and retinal pigment epithelial cell^4^ images in DL framework. Consequently, the DL network successfully applied in plant biology for stomata classification, detection^5–7^, and counting^8^, plant protein subcellular localization^9^, xylem vessels segmentations^10^, and plant cell segmentation for automated cells tracking^11,12^. Therefore, it is necessary to assemble a large number of annotated microscopy images and its ground truth for the successful application of DL based microscopy image analysis^13^. Certainly, there are numerous publicly available microscopy image datasets, mostly medical images for DL based diagnosis and prognosis, such as Human Protein Atlas^14^, H&E-stained tissue slides from the Cancer Genome Atlas^15^, DeepCell Dataset^16,17^, Mitosis detection in breast cancer^18^, lung cancer cell^19^. In contrast, there are very few number of publicly accessible biological microscopy image datasets of plant tissue cells, which are suitable for the DL framework. Furthermore, the existing datasets have limited number of diverse images with proper annotation. In such context, we have generated an optical microscopy image dataset of potato tuber with a larger number of diverse images, and appropriate annotation. This publicly available dataset will be beneficial in analysing the plant tissue cells with great details by employing DL based techniques.

Microscopy image analysis has become more reliable in understanding the structure, texture, geometrical properties of plant cells and tissues which pay a profound impact on botanical research. Such studies have significant aspects in interpreting the variety of different plant cells, tissues, and organs by discriminating cell size, shape, orientation, cell wall thickness, distribution, and size of intracellular spaces^20^, tissue types, and mechanical^21,22^ properties like shear, compressive stiffness etc. For instance, the shape and size of cell guides to determine the size and texture^23^ of a plant organ; while, the tissue digestibility and plant productivity^24,25^ are controlled by the cell wall thickness; similarly, the mechanical properties of the cell wall plays a crucial role in plant stability and resistance against pathogens^26^; whereas, the intercellular spaces influence the physical properties of tissues, like firmness, crispness, and mealiness^27^. Certainly, it has been practiced in various domains of plant cell research, such as fruits and vegetables^23,28^. In this connection, there are various ways to generate microscopy images, such as brightfield microscopy, fluorescence microscopy, and electron microscopy. All these methods have their own advantages and disadvantages as well. Besides, sample preparation is one of the crucial steps in microscopy image generation which includes fixation, paraffin embedding, and different staining techniques for better visualization of cell segments. The most widely used stains are safranin-o^29,30^ and toluidine blue-o^31^ for visualizing cell walls and lugol’s iodine^32^ for starch detection.

In this view, we present a large brightfield optical microscopy image dataset of plant tissues of potato tuber, as it is one of the principal and high productive tuber crops and a valuable component of our regular diet. Usually, potato tubers are of oval or round shape with white flesh and pale brown skin with bud and stem end. Three major parts of the tuber are cortex, perimedullary zone (outer core), and pith (inner core) with medullary rays, which are made up of parenchyma cells. The cell structures are distinct for different tuber variety^33^, even within the same tuber, especially inner core and outer core^34^. The same structural differences can also be observed between the stem and bud ends. In addition, the cell division and enlargement in various regions play an important role^35^ on potato tuber growth. Following such variations in cell structure, we have generated a large dataset consisting of 15,938 fully annotated unstained and stained images with three-fold labelling. The labelling has been prepared based on the tuber size (large, medium, and small), collections area (bud, middle and stem part), and tissue zones (inner and outer core) and the images have been graded as physical, morphological and tissue grading respectively. In addition, 60 ground truth segmentation labels of the images from the inner core have been prepared for the different tuber weight. To check the quality of the images, technical validation has been conducted by the DL based classification and segmentation tasks, which displayed significant recognition accuracy. Thus, this dataset is very much suitable for studying plant cell microstructures including cell size and shape, cell wall thickness, intercellular space, starch, and cell density distribution in potato tubers using DL based pipeline. Indeed, such properties can be explored explicitly as the dataset includes the images from the entire region of the tuber covering two tissue zones from stem to bud end for different tuber weights. In addition, large number of images in this dataset will provide new opportunities for evaluating and developing DL based plant biology classification and segmentation algorithms. Furthermore, the unstained along with stained images will be suitable to develop virtual-staining algorithms in the DL framework. Therefore, the dataset could enrich the DL based microscopy cell assessment in plant biology substantially.

## Methods

### Potato tuber selection and microscopic specimen preparation

The raw potato tubers (Solanum tuberosum L.) of an Indian variety, Kufri Pukhraj have been chosen in this work. The Kurfi Pukhraj, an excellent source of vitamin C, potassium, and fibre is one of the popularly grown commercial cultivars in India. The tubers have been collected immediately after harvesting in mid of December 2019 from Kamrup, a district of Assam state, India. All the samples without any outer damages have been collected and stored in the temperature of 19.2 °C–29.2 °C with 70% relative air humidity. Based on the weight of the tuber, samples have been graded into large, medium, and small of weight 80–100 gm, 40–50 gm, and 15–25 gm respectively. From each of these groups, 5 samples (total of 15 samples) have been selected for image generation at the laboratory maintaining stable room temperature and humidity. The whole experiment including collection of the tuber samples and image generation has been accomplished in 20 days. Different graded tuber samples are chosen alternate days during the experiment.

**Fig. 1:**
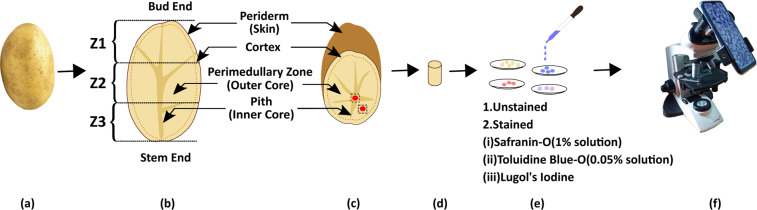
Demonstration of potato tuber anatomy, sample preparations, and image acquisition set up for microstructure visualization: (**a**) A potato tuber sample. (**b**) Longitudinal cross-section of a tuber. The samples have been divided into three parts, named Z1, Z2, and Z3 nearer to bud, middle and stem respectively as indicated by dotted lines for microscopic observations. (**c**) Transverse cross-sections of the tuber where sample collection areas, inner and outer core are highlighted by red circle. (**d**) Tissue samples have been collected by using a cork borer of diameter 4 mm from specified zones. (**e**) Thin free-hand unstained sections have been obtained. The stained samples have been prepared by using safranin-o (1%), toluidine blue-o (0.05%), and lugol’s iodine. (**f**) Image capturing set up in which, the camera of the smartphone has been fixed on the microscope eyepiece by using an adaptor. Two types of microscopy images, unstained and stained images have been captured independently without drying the sections.

The major parts of potato tuber, periderm (skin) with the bud and stem ends, cortex, perimedullary zone (outer core), and pith (inner core) with medullary rays have been displayed in Fig. 1b,c. The periderm, the outermost layer, protects a tuber from dehydration, infection, and wounding during harvest and storage. The cortex, outer core, and inner core tissues appear successively after the skin where starch granules are stored in parenchyma cells. The thickness of the cortex is about 146–189 µm^36^ and the largest cells are found here. The outer core spreads about 75% of the total tuber volume and contains the maximum amount of starch^37^. The innermost region i.e., inner core expands from stem to bud end^38^ along longitude direction; whereas, the medullary rays spread toward the cortex. The samples have been collected from the inner and outer core which covers most of the areas of a tuber. Besides, the cell structures^34^ and the amount of starch are distinct in these two tissue zones. Similar samples have been collected from three areas, named Z1, Z2, and Z3 as indicated in Fig. 1b. The samples have been extracted with a cork borer of a diameter of 4 mm and rinsed in distilled water. After that, 5 thin sections from the inner core as well as the outer core of each of the three areas have been collected. Therefore, from a tuber sample, 30 thin sections (5 sections ×3 section areas ×2 tissue zones) have been analysed. Furthermore, to capture images, fresh thin potato sections (i.e., unstained samples) have been placed under the microscope. In addition, for the better visualization of cell boundaries and subcellular components, especially starch, the samples have been stained. Safranin-o (1% solution) and toluidine blue-o (0.05% solution) has been used to visualize all cell walls; whereas, lugol’s iodine solution helped to distinguish starch granules. An optical microscope (Labomed Lx300, Labomed America) accompanied by a smartphone camera (Redmi Note 7 Pro) was used to generate and capture microscopy images as shown in Fig. 1f. The brightfield microscopy images have been generated using a 10x lens (field number = 18, numerical aperture = 1.25) which provides a field of view (FOV) of diameter 0.18 mm. The camera of the smartphone has been fixed on the microscope eyepiece by using an adaptor. Certainly, the exposure and white balance state has been secured by the adequate brightness level of the microscope’s built-in light-emitting diode (LED) and a clear FOV. The exposure time of the smartphone camera has been kept in the range of 1/200 s– 1/100 s which provides satisfactory brightness level; whereas, the focus setting of the camera has been locked that maintains fixed magnification among all the images. The images have been captured in the highest quality JPG format with maximum of 10% compression only to retain the image quality reasonably high. The mobile camera has been fixed to 3x zoom which offers a FOV of 890 × 740 µm^2^ with an approximate resolution of 0.26 µm/pixel. Following this setting, three images have been taken for each field of view by changing the focus distance of 3 µm. Similarly, around 15 images have been acquired from a section by continuous precision shifting of the microscope stage along the x-y plane before the samples get dried. Thus, in total 9,811 unstained and 6,127 stained images have been captured and saved in JPG format in 24-bit RGB color and of resolution 3650 × 3000.

### Image grading

**Fig. 2:**
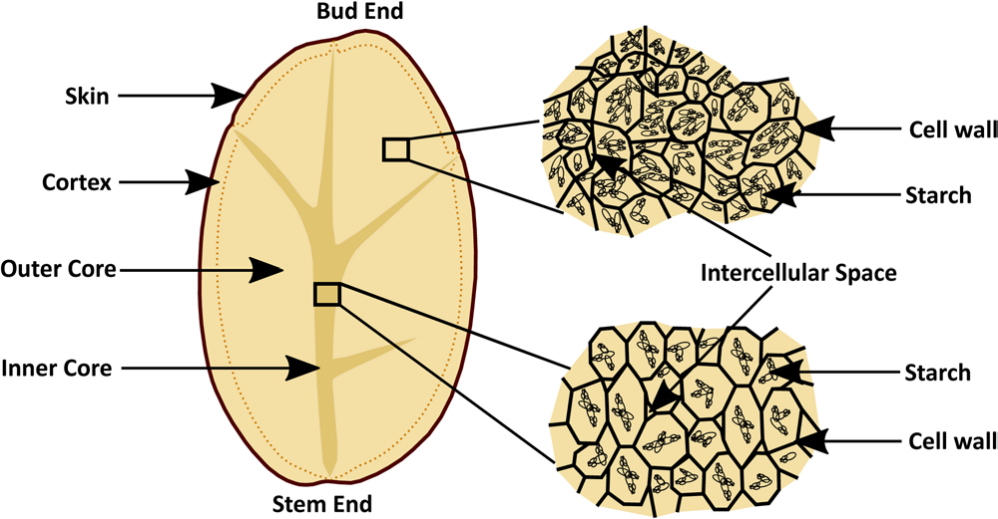
A schematic diagram displaying inner and outer core cell characteristics of a potato tuber.

Previous studies identified that the potato tuber weight is directly associated with the number of cells and cell volume in different tissue zones. Nevertheless, the cell numbers are considered as a significant factor compared to mean cell volume for a tuber weight variation^39^. Hence, potato tuber weight has been recognized as one of the important physical parameters to achieve versatility in the image database. Therefore, in this work, based on the weight, potato tubers are categorized into three groups as large, medium, and small. Certainly, the captured microscopic images are composed of discrete cells with thin nonlignified cell walls surrounded by starch granules^40^. In a tuber, the cell size differs considerably in the two tissue zones— inner and outer core^34^. In general, the outer core occupies the maximum volume of the tuber and stores the largest number of starch granules as reserve material. On the contrary, the inner core cells are smaller^34^ with lower starch content which makes this tissue zone wet and translucent as displayed in Fig. 2. Such variation of cell sizes and starch distribution can be observed in the stem, bud, and middle section of tubers as well. Therefore, the images have been graded into three categories namely (1) physical grading, (2) morphological grading and, (3) tissue grading based on tuber weight, section areas, and tissue zones respectively.

#### Physical grading

Tubers of three different weight ranges have been selected for the image dataset, as it has a correlation with the cell features. Three different weight groups of tubers, such as large (L), medium (M), and small (S) with weight 80–100 gm, 40–50 gm and 15–25 gm respectively, have been considered for this microscopy image dataset. The generated images have been labelled with L, M, and S followed by sample number 1–5 to distinguish tuber weight along with sample number; for instance, L1 refers to the first sample of a large tuber. The labels associate with weights and related parameters along with sample numbers for physical grading have been listed in Table 1.

**Table 1:** Summary of different parameters for physical grading including weight and associated measures of potato tubers.

SI. No.	Weight Categories	Label	Weight (gm)	Length (mm)	Width (mm)	Thickness (mm)	Sample’s No.
1	Large	L	80–100	65–80	50–55	40–45	1–5
2	Medium	M	40–50	50–65	40–45	30–40	
3	Small	S	15–25	38–50	25–35	25–30	

#### Morphological grading

The bud and stem ends of potato tubers are connected with the apical and basal end of the shoot respectively. These areas displayed compositional variations^41,42^ with distinct cell features. The images of the tuber middle part (separates the bud, and stem end) have been incorporated in this dataset to visualize structural variations along the longitudinal direction. Therefore, for morphological grading, the tubers have been divided into three parts namely Z1, Z2, and Z3 which specify the bud, middle, and stem areas respectively as shown in Fig. 1b. Certainly, the images have been captured from these areas for each physically graded sample and labelled accordingly.

#### Tissue grading

**Fig. 3:**
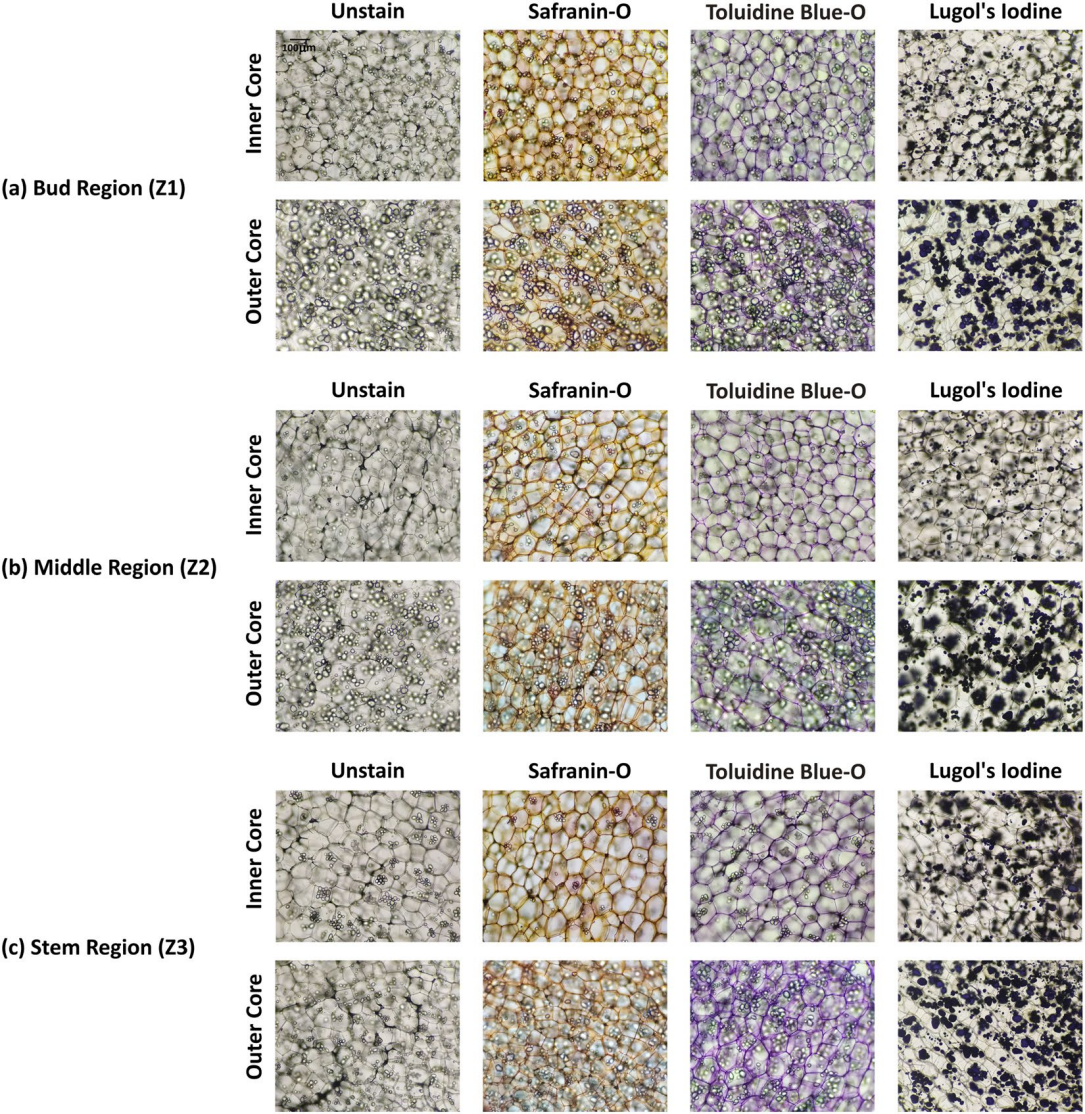
Example of unstained and stained images of large (80–100 gm) potato tubers. Rows and columns indicate respective tissue zones (inner and outer core) and different staining agents. The first column specifies the unstained images, whereas, the subsequent columns are for stained images of safranin-o, toluidine blue-o, and lugol’s iodine. The images are from (**a**) Bud Region (Z1), (**b**) Middle Region (Z2), and (**c**) Stem Region (Z3).Note: All the images are with the scale at top-left corner on unstained image.

**Fig. 4:**
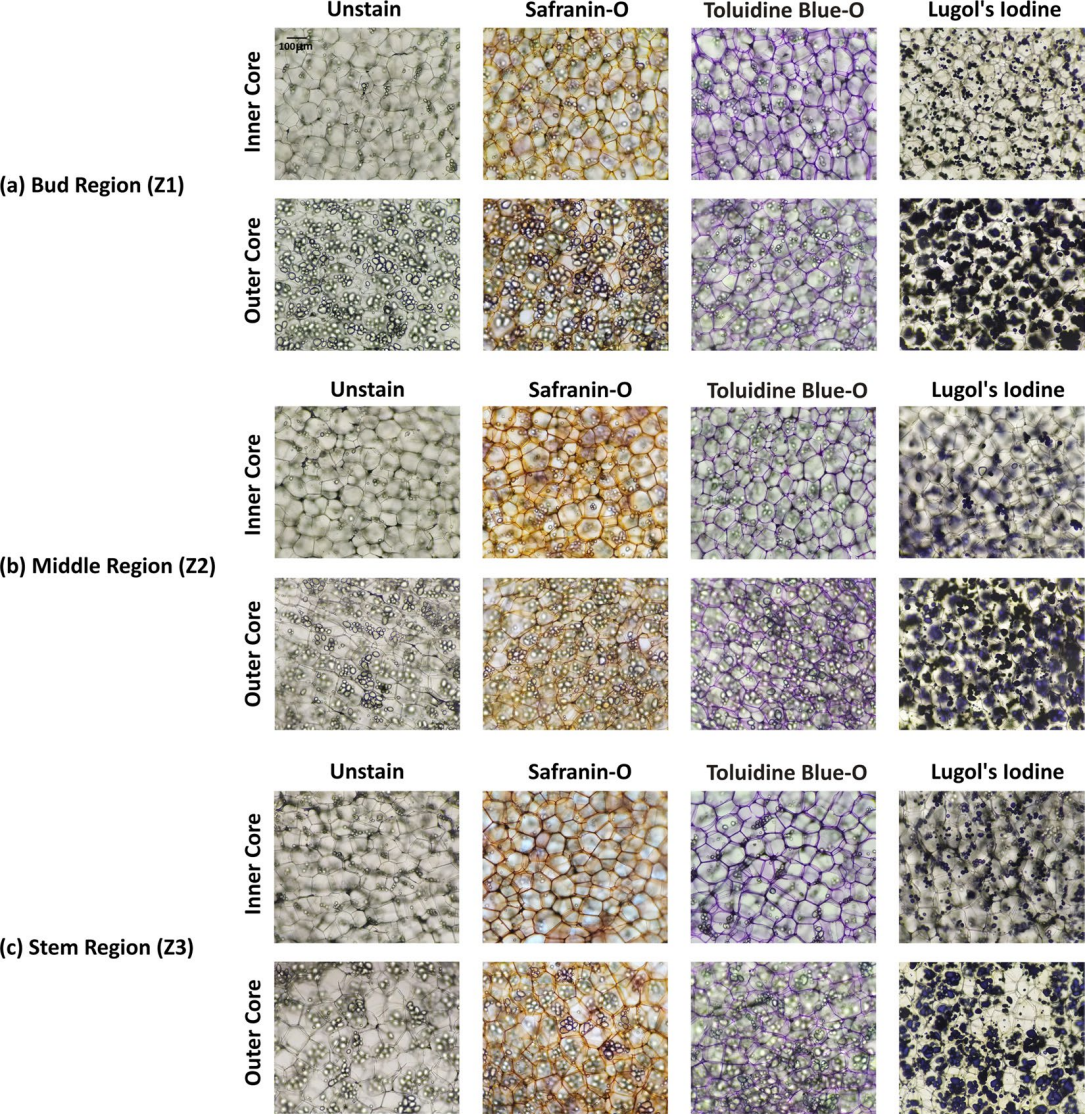
Example of unstained and stained images of medium (40–50 gm) potato tubers. Rows and columns indicate respective tissue zones (inner and outer core) and different staining agents. The first column specifies the unstained images, whereas, the subsequent columns are for stained images of safranin-o, toluidine blue-o, and lugol’s iodine. The images are from (**a**) Bud Region (Z1), (**b**) Middle Region (Z2), and (**c**) Stem Region (Z3). Note: All the images are with the scale at top-left corner on unstained image.

**Fig. 5:**
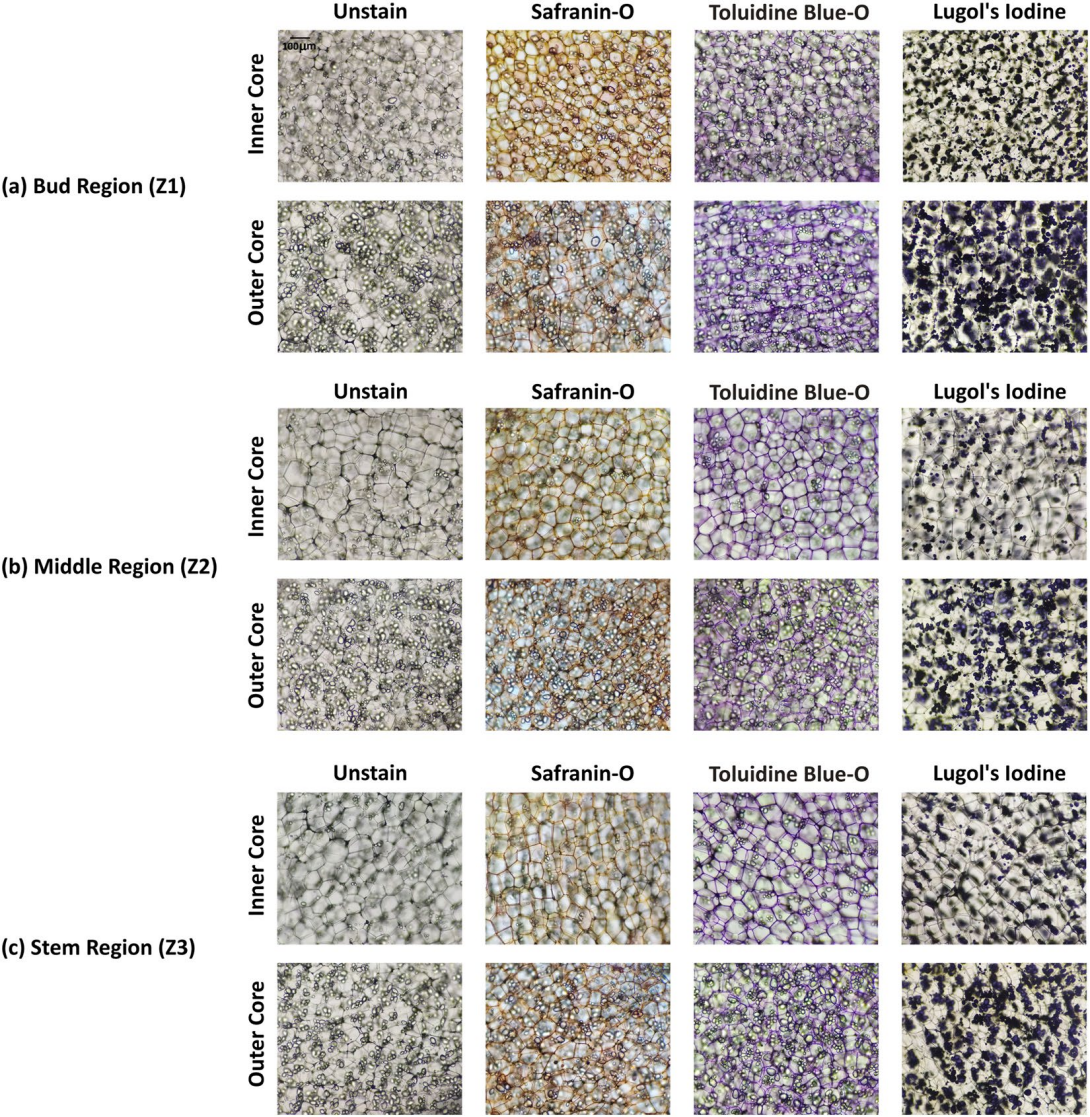
Example of unstained and stained images of small (15–25 gm) potato tubers. Rows and columns indicate respective tissue zones (inner and outer core) and different staining agents respectively. The first column specifies the unstained images, whereas, the subsequent columns are for stained images of safranin-o, toluidine blue-o, and lugol’s iodine. The images are from (**a**) Bud Region (Z1), (**b**) Middle Region (Z2), and (**c**) Stem Region (Z3). Note: All the images are with the scale at top-left corner on unstained image.

A significant variation in cell sizes within the same potato tuber can be observed in inner core and outer core tissue zones. The cell size of the outer core is larger than that of the inner core and contains most of the starch material. Therefore, in tissue grading, these two zones have been identified. Certainly, the images have been captured from these zones for each morphologically graded sample and labelled as IC and OC which indicates the inner and outer core of the potato tuber respectively. Example of unstained and stained images of large, medium, and small potato tuber from different section areas and tissue zones have been displayed in Figs. 3–5 respectively.

## Database Summary

There are total 15,938 (9,811 unstained and 6,127 stained) numbers of images in this dataset. The images are categorized based on different grading and labelling basis, and listed in Table 2. The first two columns refer to grading and labelling basis followed by the number of images for unstained and stained cases. Furthermore, the stained images with three stains (safranin-o, toluidine blue-o, and lugol’s iodine) are also specifically mentioned.

**Table 2:** The number of images under each grading for unstained and stained image dataset.

Grading Basis	Labelling Basis	Number of Images			
		Unstained	Stained		
			Safranin-O	Toluidine Blue-O	Lugol’s Iodine
Physical	Large (L)	3,311	674	683	680
	Medium (M)	3,200	671	663	653
	Small (S)	3,300	702	705	696
Morphological	Bud (Z1)	3,271	708	690	655
	Middle (Z2)	3,327	660	698	674
	Stem (Z3)	3,213	679	663	700
Tissue	Inner Core (IC)	5,220	1,079	1,042	993
	Outer Core (OC)	4,591	968	1,009	1,036

### Ground truth label generation for cell boundary segmentation

Segmentation is performed to split an image into several parts to identify meaningful features or objects. In microscopy image analysis, a common problem is to identify distinct parts which correspond to a single cell or cell components to quantify the spatial and temporal coordination. Furthermore, as a precursor to geometric analysis, such as cell size and its distributions, image segmentation is essential. Such a task can be performed manually, which is very much time-consuming, irreproducible, and tedious for larger image sets. Nonetheless, it can be automated by the ML techniques which require proper ground truth labels. Therefore, we have generated ground truth labels of cell boundaries for the automated segmentation task. The images have been captured from different parts of the tubers as mentioned earlier, and labelled accordingly. Certainly, to generate the ground truth labels for cell boundary segmentation, the unstained images of inner core from the Z2 area have been selected, as cell boundaries are comparatively prominent in this zone due to presence of fewer amounts of starch granules.

**Fig. 6:**
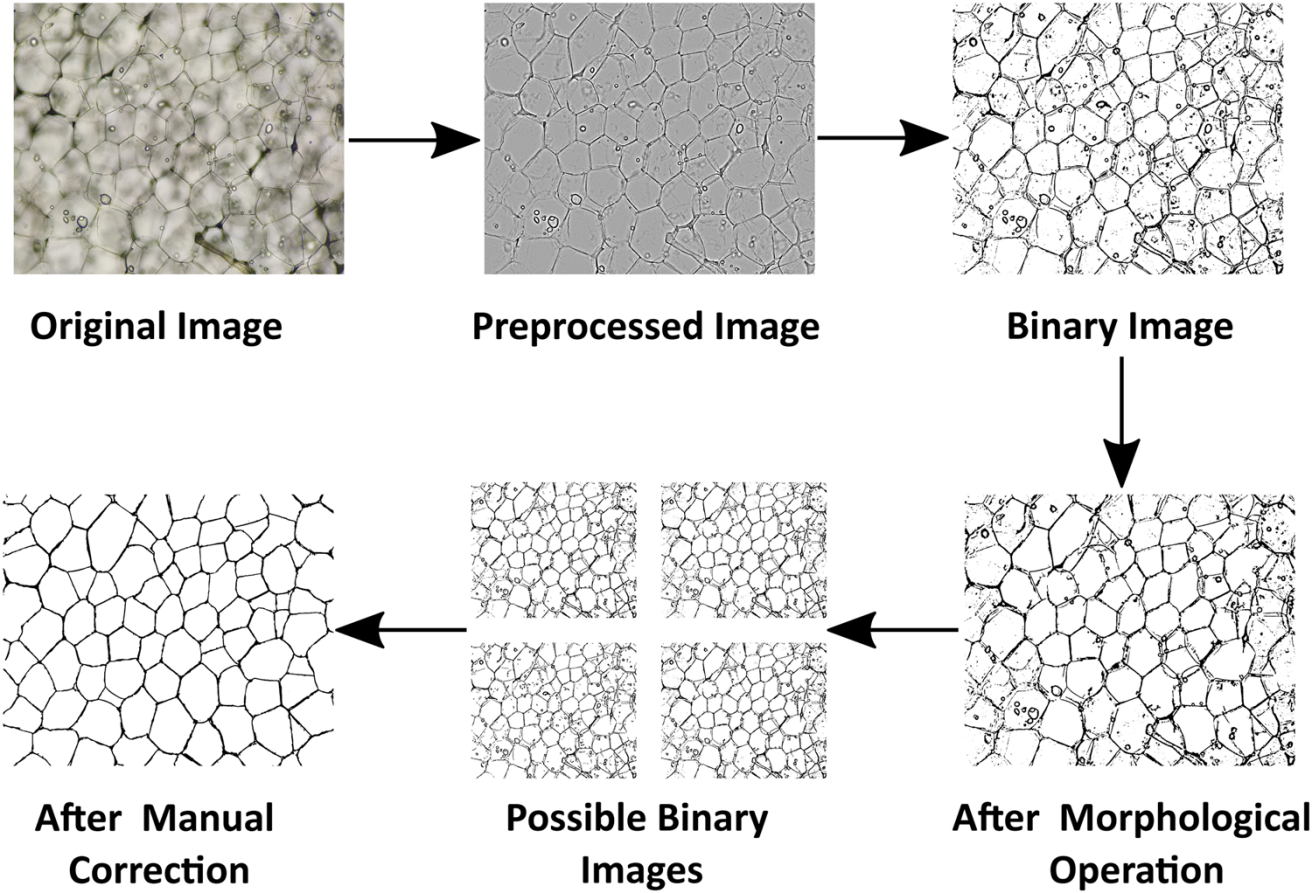
Steps involved in generating the ground truth segmentation labels for the inner core tissues. The original images pre-processed by employing rolling ball algorithm and bandpass filtering. Next, the adaptive thresholding has been employed to obtain binary images. Furthermore, morphological operations have been performed to refine the cell boundaries and remove the starch granules. By changing *f*_*cl*_, *f*_*ch*_ and *R* at pre-processing steps, possible binary images have been generated. Then, the best image has been selected for manual correction.

Segmentation of potato cell images can be very much challenging because of its complex cell boundaries and non-uniformity in image background which leads to poor contrast between cell boundary and background. Therefore, to generate the ground truth cell boundaries, a few steps have been involved: (1) pre-processing (2) thresholding, and (3) morphological operations. The pre-processing steps have been mainly implicated in background correction and image filtering. Generally, the uneven thickness of the tuber section results non-uniform microscopy image background. Thus, to minimize such non-uniformity a well-known rolling ball algorithm^43,44^ has been employed. It eliminates the unnecessary background information by converting a 2D image *I*(*x*,*y*) into a 3D surface; where, the pixel values are considered as the height. Then, a ball of a certain radius (*R*) is rolled over the backside of the surface which creates a new surface *S*(*x*,*y*). Furthermore, a new image with a uniform background is created by, ${I}_{new}(x,y)=(I(x,y)+1)-(S(x,y)-R)$^44^. To achieve an optimal image with the best uniform background, the values of *R* must be selected carefully. In our work, empirically, the values of *R* have been kept as 30 < *R* < 60. Next, for image filtering, bandpass filter has been used to enhance the cell edges by eliminating shading effects. In this purpose, Gaussian filtering in Fourier space has been considered. A bandpass filter having two cut-off frequencies, lower (*f*_*cl*_) and higher (*f*_*ch*_) are kept within a range for intensity variation in the captured image. Empirically, it has been kept as 10 < *f*_*cl*_ < 30 and 60 < *f*_*ch*_ < 120. Furthermore, the adaptive thresholding method^45^ has been implemented to binarize the images for discriminating the cell boundaries. Moreover, morphological operators, such as opening, closing, and hole filling has been chosen to refine cell boundaries. Several values of *f*_*cl*_, *f*_*ch*_, and *R* have been chosen to get the best binary images. Although, very few starch granules and some disconnected cell boundaries can be observed in the resultant binary images, which could lead to a weak cell boundary segmentation. Certainly, such discrepancies have been further refined by very well-known manual process^46^ which involves removal of the starch granules and contouring cell boundaries. The whole process of cell boundary segmentation ground truth label generation has been shown in Fig. 6.

## Data Records

This dataset is publicly available on *figshare*^47^ (10.6084/m9.figshare.c.4955669) which can be downloaded as a zip file. The zip file contains three folders named as “stain”, “unstain”, and “segmentation”. All the images are in JPG format. The raw microscopy images of potato tubers can be found in “stain” and “unstain” folder; whereas, the segmentation folder provides raw images with ground truth segmentation labels. The “stain” folder contains different stained images in the respective folders named as “safranin”, “toluidine”, and “lugols”. The image labels information can be extracted from the image filenames itself. The file naming format for unstained image is as < physical grading with sample no. > _ < morphological grading > _ < tissue grading > _ < section no. > _ < image no. > ; for example, M1_Z1_IC_Sec. 1_02.jpg refers to an image (first section out of five) taken from the inner core of Z1 of medium weight potato tuber (sample no. 1). Similarly, the stained image file naming format is as < physical grading with sample no. > _ < morphological grading > _ < tissue grading > _ < section no. > _ < stain type > _ < image no.>; for example, S1_Z2_OC_Sec. 3_lugol_02.jpg refers to an lugol’s iodine stained image (third section out of five) taken from the outer core of Z2 of small weight potato tuber (sample no. 1). The whole file naming format can be understood by following Table 3. The segmentation folder contains two subfolders, named as “images” and “groundtruth”. The image files naming format is as < physical grading > _ < image no. >; for example, “L_2.jpg” represents the second image of a large potato tuber sample. Besides, the ground truth label images are kept in binary image format having the same dimensions of the raw images.

**Table 3:** Raw image file name format.

Physical Grading	Sample No.	Morphological Grading	Tissue Grading	Section No.	Stain Type	Image No.	Remark
Large (L)	1 to 5	Bud (Z1)	Inner Core (IC)	Sec <1 to 5>	Safranin-o (safo) Toluidine blue-o (tolu) Lugol’s iodine (lugol)	n	L1_Z1_OC_Sec. 1_1
							L1_Z1_IC_Sec. 4_safo_1
Medium (M)		Middle (Z2)					M1_Z1_OC_Sec. 1_2
			Outer Core (OC)				M2_Z1_IC_Sec. 1_tolu_1
Small (S)		Stem (Z3)					S2_Z2_OC_Sec. 3_3
							S1_Z1_IC_Sec. 3_lugol_3

## Technical Validation

**Fig. 7:**
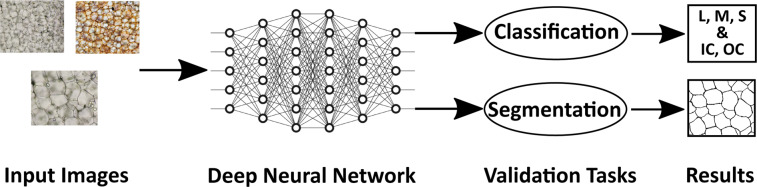
Overall technical verification of image and ground truth segmentation label. Two types of microscopy images have been chosen independently. The image labels have been verified by VGG16^48^ deep neural network employing transfer learning. The Unet^50^ architecture has been used to employ the semantic segmentation using the generated ground truth segmentation labels and hence verify the same.

The technical validation has been conducted by employing the DL based classification and segmentation tasks on the acquired image dataset as illustrated in Fig. 7. Multi-label cell classification has been conducted to verify the quality of the assigned labels. It has been examined by considering two specific image labels— physical (L, M, and S) and tissue grading (IC and OC). Besides, to verify the ground truth segmentation labels, semantic segmentation has been performed using the DL pipeline. The first test can yield information about the possible separation of labels and the later can access individual cells in different tuber weights.

### Multi-label cell classification

The CNN classification network, VGG16^48^ has been employed for multi-label cell classification using input images of 256 × 256 pixels with two labels, physical (L, M, and S) and tissue grading (IC and OC). The first 13 layers of the neural network have been pre-trained on ImageNet [ILSVRC2012] dataset. On top of it, task-specific fully-connected layers have been attached and activated by the sigmoid function. The complete network has been fine-tuned on our datasets. The network performance has been evaluated based on the train-test scheme. Therefore, the entire image dataset (unstained and stained) has been partitioned randomly into two subsets, with 80% for training and 20% for test. The network has been trained using SGD^49^ optimizer with a learning rate of 10^−2^, momentum 0.9, and the binary cross-entropy as loss function for both the image dataset. With the iterative learning technique, performance metrics, such as accuracy and F2-score (assessing the correctness of the image labels), have been obtained for test images. The results have been listed for the test set in Table 4. It shows that for the same number of epochs (30), the unstained image dataset gives a better result than the stained image dataset.

**Table 4:** Performance assessment based on accuracy and F2-score of unstained and stained image dataset.

Model	Accuracy	F2-score
Unstained images as train and test	0.9427	0.9207
Stained images as train and test	0.9205	0.8918

### Cell segmentation

**Fig. 8:**
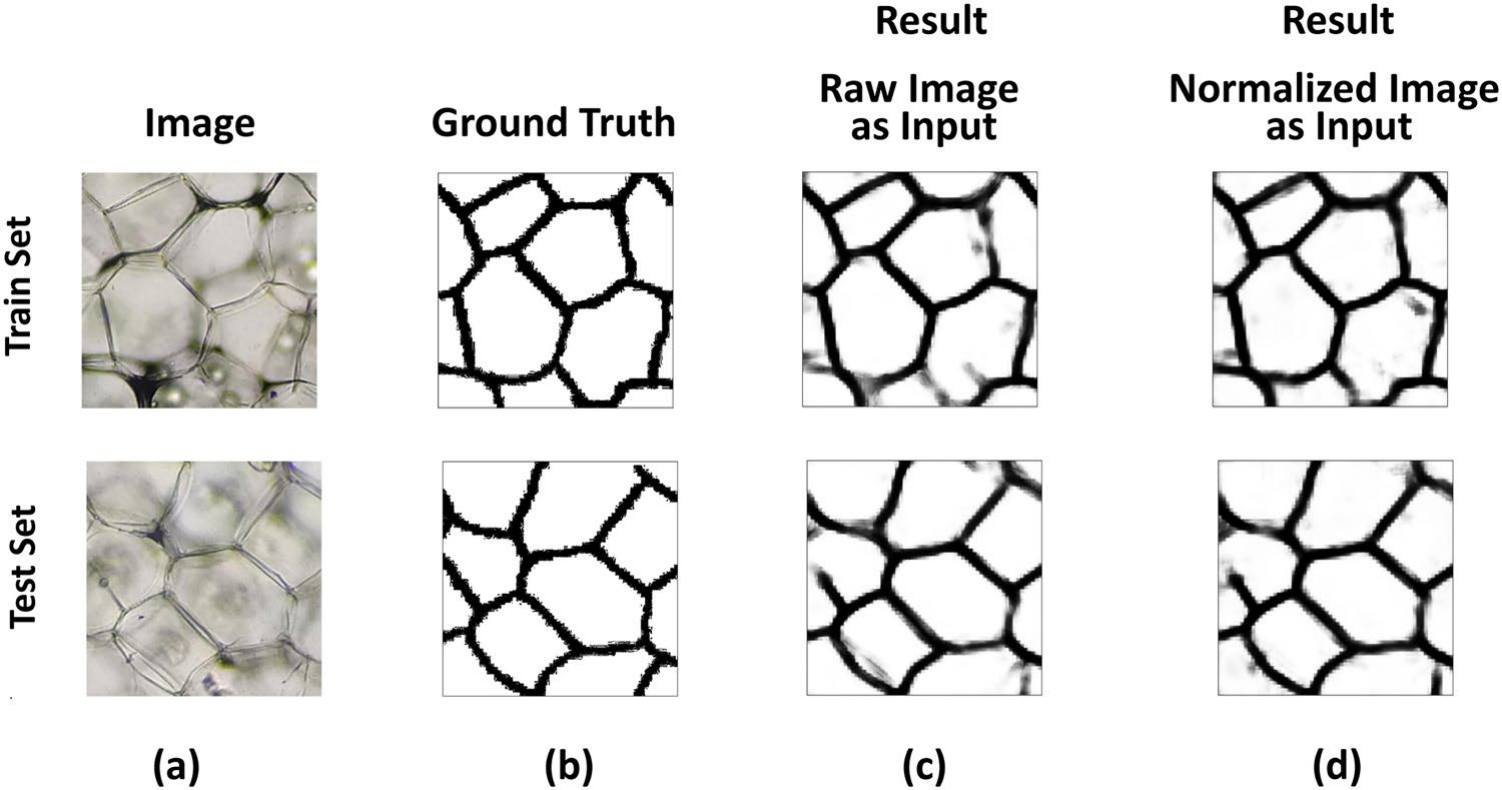
Example of different image sets used during cell segmentation by Unet^50^ for ground truth labels validation. The first and second rows indicate the train and test images respectively. (**a**) Unstained raw RGB images, (**b**) Ground Truth images, (**c**) Segmentation result for raw RGB input images, and (**d**) Segmentation result for normalized input images.

In this task, Unet^50^, a very well recognized image segmentation neural network has been employed. It has shown remarkable performance in biomedical image segmentation. The input images have been generated by subdividing each ground truth labels and raw images into 20 sub-images, which further resized to 512 × 512 pixels before training. The network has been trained using Adam^51^ optimizer with learning rate of 10^−1^. Two types of inputs, namely raw and normalized images have been given separately into the network. The entire image dataset has been partitioned randomly into two subsets, with 80% for training and 20% for test. Then, performance evaluation has been conducted by employing normal adaptive learning rate-based training. During the training period, early stopping has been used to choose the model with the highest validation performance. The mean intersection of union (IOU) has been chosen as a performance metric that measures how much predicted boundary overlaps with the ground truth (real cell boundary) and the results have been displayed in Table 5. For the same deep neural network, normalize input images give better result than the raw images. A representative result of cell segmentation for raw and normalized input images has been displayed in Fig. 8 in which (a), (b), (c), and (d) refer to raw RGB image, ground truth, the result for raw and normalized input images respectively.

**Table 5:** Performance assessment based on mean IOU of raw and normalize image dataset.

Model	Input type	Mean IOU
Cell Boundary as train and test	Raw Images	0.6964
	Normalized Images	0.7020
